# Development of antibody-dependent cellular cytotoxicity in response to recombinant and live-attenuated herpes zoster vaccines

**DOI:** 10.1038/s41541-022-00545-2

**Published:** 2022-10-25

**Authors:** Seong Yeon Park, Myron J. Levin, Jennifer Canniff, Michael Johnson, D. Scott Schmid, Adriana Weinberg

**Affiliations:** 1 Department of Infectious Diseases, Dongguk University Ilsan Hospital, Goyang, Republic of Korea; 2 Departments of Pediatrics, University of Colorado School of Medicine, Aurora, CO USA; 3 Department of Medicine, University of Colorado School of Medicine, Aurora, CO USA; 4 Centers for Disease Control and Prevention, Division of Viral Diseases, Atlanta, GA USA; 5 Departments of Pathology, University of Colorado School of Medicine, Aurora, CO USA

**Keywords:** Medical research, Translational research

## Abstract

Zoster vaccines generate antibody responses against varicella-zoster virus (VZV). We compared antibody-dependent cell cytotoxicity (ADCC) elicited by zoster vaccine live (ZVL) and recombinant zoster vaccine (RZV). ADCC mediated by antibodies against VZV lysate (VZV-ADCC) and recombinant glycoprotein E (gE-ADCC) was measured using plasma from 20 RZV- and 20 ZVL-recipients, including half 50–60-years-old and half ≥70-years-old. Solid phase-bound anti-VZV antibodies stimulated TNFα in NK cells as measured by flow cytometry or ELISA. VZV-ADCC pre- and post-immunization was higher in younger vaccinees. ZVL did not appreciably increase VZV-ADCC, whereas RZV increased VZV-ADCC in older vaccinees. ELISA-measured gE-ADCC was similar across groups pre-immunization; significantly increased after ZVL; and RZV and was higher in younger RZV than ZVL recipients. IgG3 antibodies increased after RZV and ZVL, with greater anti-gE than anti-VZV responses. Moreover, gE-ADCC strongly correlated with anti-gE antibody avidity, but there were no appreciable correlations between VZV-ADCC and avidity. NK cells stimulated by anti-gE antibodies showed increased IFNγ and CD107a expression, which was not observed with anti-VZV antibodies. In conclusion, anti-gE antibodies generated more robust ADCC than anti-VZV antibodies. RZV induced higher ADCC antibodies than ZVL depending on the antigen and age of vaccinees. Older adults had lower ADCC antibodies before and after vaccination than younger adults.

## Introduction

Herpes zoster (HZ), which is caused by reactivation of varicella-zoster virus (VZV) latent in cranial nerve and sensory ganglia following varicella, is prevented by the VZV-specific immune responses that develop during childhood varicella^1^. Abundant clinical and immunologic data indicate: that VZV-specific cell-mediated immunity (CMI) is necessary and sufficient to prevent HZ^2–4^; that the frequency and severity of HZ correlates inversely with these responses^2,4^; and that there is no such correlation with VZV-specific antibody^2,5,6^. However, such antibody(ies) could contribute to the prevention or recovery from HZ. For example, antibody responses after live HZ vaccine (ZVL, Zostavax, Merck) correlated with protection^2^. In another study with ZVL the fold-rise in the antibody response qualified as a surrogate marker for protection^7^. However, a mechanism involving antibody in protection against HZ remains undefined. For example, it is unlikely that neutralizing antibodies play a role in protection against VZV, which spreads readily from cell-to-cell and which circulates within immune cells^8,9^. Two other mechanisms have been proposed by which VZV-specific antibody might limit VZV infection in general, and HZ in particular. The first proposal is that the antibody attaches to and interferes with the function of a virally encoded fusogen within the cell membrane, thus inhibiting virion egress and cell-to-cell spread or infectivity^10^. This mechanism is based on in vitro model that has not been universally reproduced. A second, more plausible role for VZV-specific antibodies, is antibody-dependent cellular cytotoxicity (ADCC). It is well established that individuals with defects in NK cells, which are central to ADCC, suffer from severe and recurrent, often fatal VZV infections^11,12^. ADCC may also contribute to the partial clinical success achieved using high titer VZV hyperimmune globulin for passive immunization of naïve individuals^13,14^. Investigations of ADCC mediated by VZV antibodies were undertaken several decades ago with the methods then available^15–18^. In this report we apply contemporary methods to determine if ADCC antibodies are stimulated by the two licensed HZ vaccines – the live attenuated vaccine, ZVL and the adjuvanted recombinant glycoprotein E (gE) zoster vaccine (RZV). This is critical in understanding the basis of the striking differences in efficacy and applicability of these vaccines to an elderly population^19^. Differences in CMI in response to each vaccine have been demonstrated^20^. The current report investigates whether functional differences in the humoral response to the two vaccines might contribute to differences in their efficacy.

## Results

### Solid phase bound anti-VZV antibodies stimulate ADCC measured by TNFα using flow cytometry or ELISA

**Fig. 1:**
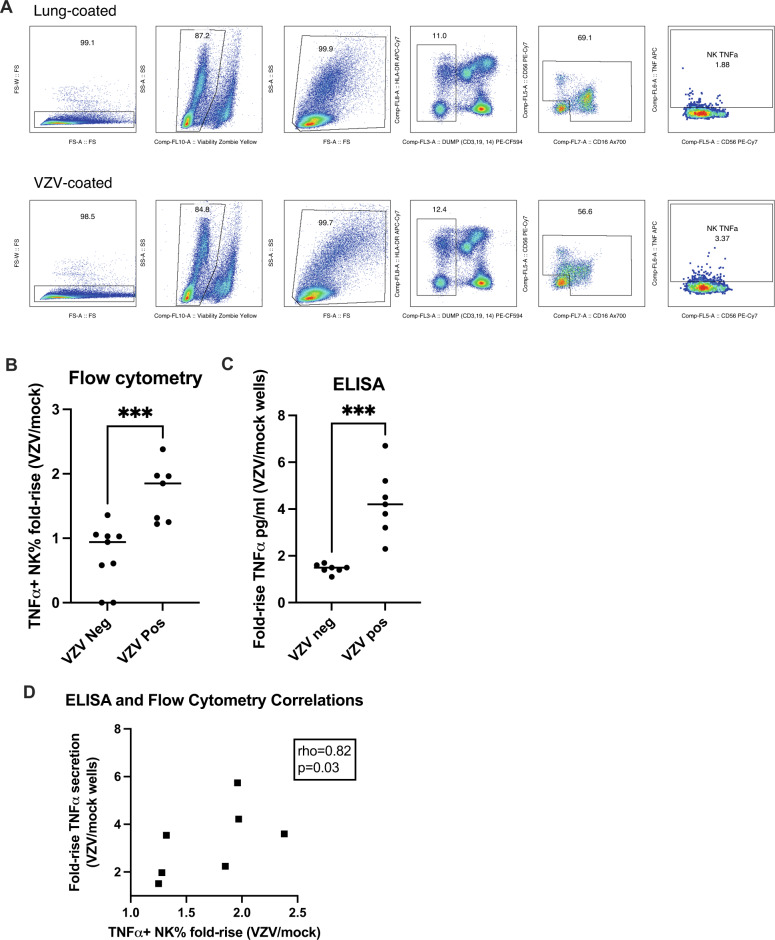
VZV-Specific ADCC measured by the NK cell production of TNFα. Data were derived using 9 VZV-seronegative and 7 VZV-seropositive serum samples and PBMC from a single leukopack. **A Gating strategy** of the flow cytometry assay. **B Flow cytometry results**. Data points indicate the proportions of TNFα-secreting NK cells in wells coated with VZV antigen/wells coated with mock-infected control antigen. The horizontal lines indicate the means. *** indicate *p* = 0.001 by unpaired T test. **C ELISA results**. Data points represent the proportions of TNFα-secreting NK cells in wells coated with VZV antigen/wells coated with mock-infected control antigen. The horizontal lines represent the means. *** indicate *p* = 0.0002 by unpaired T test. **D Correlation between flow cytometric and ELISA results in VZV-seropositive samples**.

The assays were developed using a convenience sample of sera from 7 VZV-seropositive and 9 VZV-seronegative individuals characterized at the CDC. The ADCC methods were first validated for VZV and subsequently for gE as described in the Methods. For flow cytometry, NK cells in PBMC exposed to sera from VZV-seropositive donors bound to wells coated with VZV showed significantly higher expression of TNFα compared to PBMC exposed to VZV-seropositive sera in wells coated with mock-infected control antigen (median ratio of 1.9; *p* = 0.02, single sample Wilcoxon test; Fig. 1B). In contrast, NK cell expression of TNFα did not differ between wells coated with VZV or mock-infected control antigen when the PBMC were exposed to VZV-seronegative sera (median ratio of 1; Fig. 1B). These results indicated that the combination of VZV antibodies and VZV antigen in the wells was essential for the NK cell stimulation through ADCC. We also measured the proportion of monocytes and non-NK lymphocytes expressing TNFα in parallel with the NK cells and did not observe significant differences in their TNFα expression after exposure to VZV-seropositive compared with VZV-seronegative sera (Supplementary Fig. 1).

For the ELISA-based measure of ADCC (VZV-ADCC), we determined the amount of TNFα produced by PBMC in the conditions described above. PBMC exposed to VZV-seropositive sera generated significantly higher concentrations of TNFα in wells coated with VZV compared with wells coated with mock-infected antigen (median ratio of 4.2; Fig. 1C). In contrast, PBMC incubated with VZV-seronegative sera showed a significantly lower median ratio of 1.5 (*p* = 0.0006, Mann-Whitney test; Fig. 1C), indicating that the combination of VZV antibodies and VZV was essential for the PBMC production of TNFα through ADCC. Moreover, the proportion of NK cells measured by flow cytometry that produced TNFα when exposed to VZV-seropositive sera significantly correlated with the amount of TNFα measured by ELISA in the same experimental conditions (rho = 0.82; Fig. 1D), thus *validating* the *use of the ELISA-based assay to measure ADCC*.

### RZV and ZVL increase VZV- and gE-ADCC in vaccine- and age-dependent fashions

**Fig. 2:**
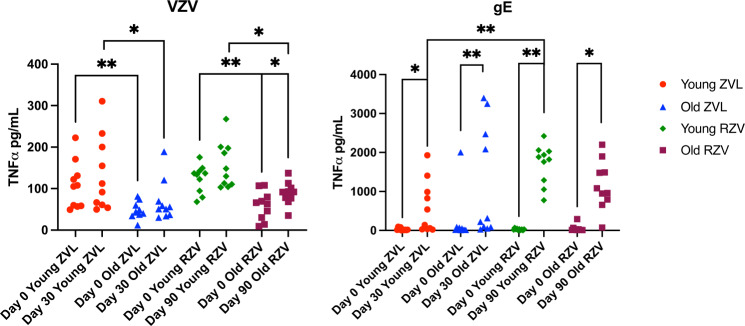
Effect of Zoster Vaccines on VZV-Specific ADCC. Data were derived from 40 individuals equally distributed between young and old ZVL or RZV recipients. Shown are TNFα concentrations measured by ELISA before vaccination and at peak response to each vaccine against VZV (**left panel**) and gE (**right panel**). Bars indicate medians and quartiles. Significant differences are shown on the graph and marked by *0.01 < *p* < 0.05 or **0.001 < *p* < 0.01.

VZV-ADCC was determined using sera obtained after RZV and ZVL administration to 10 younger and 10 older vaccine recipients (Fig. 2). VZV-ADCC was higher in younger compared with older adults pre-vaccination (medians of 106 versus 43 TNFα pg/ml, *p* = 0.007 for ZVL; and 135 versus 65 pg/ml, *p* = 0.002 for RZV; Mann Whitney U test). Similar age differences were observed for peak responses after vaccination (102 versus 53 pg/ml for ZVL at 30 days after vaccination, *p* = 0.04; and 139 versus 92 pg/ml for RZV at 30 days after the 2^nd^ dose of vaccine, *p* = 0.03). Neither ZVL nor RZV generated significant increases in VZV-ADCC in younger adults from pre- to post-vaccination. However, older ZVL recipients showed a trend toward an increase from pre-vaccination to peak response (43 versus 53 pg/ml; *p* = 0.08; Wilcoxon matched-pairs signed rank test), while older RZV recipients showed significant increases (65 versus 92 pg/ml; *p* = 0.03). The comparison of post-immunization VZV-ADCC between the two vaccines did not reveal significant differences.

gE-ADCC prevaccination was similar across age and vaccine groups (medians of 14 to 27 pg/ml; Fig. 2). Both vaccines generated significant increases at peak response in younger and older adults with medians of 340 and 269 pg/ml in younger and older ZVL recipients, and larger medians of 1853 and 1022 pg/ml in younger and older RZV recipients. The comparison of peak responses between age groups showed no difference in ZVL recipients and a trend toward higher responses in younger versus older RZV recipients (*p* = 0.07, Mann-Whitney test). The comparison of responses between vaccines showed significantly higher peak responses in younger RZV recipients compared with younger ZVL recipients (*p* = 0.002).

### RZV and ZVL preferentially increase anti-gE IgG3 compared with anti-VZV IgG3 antibodies

**Fig. 3:**
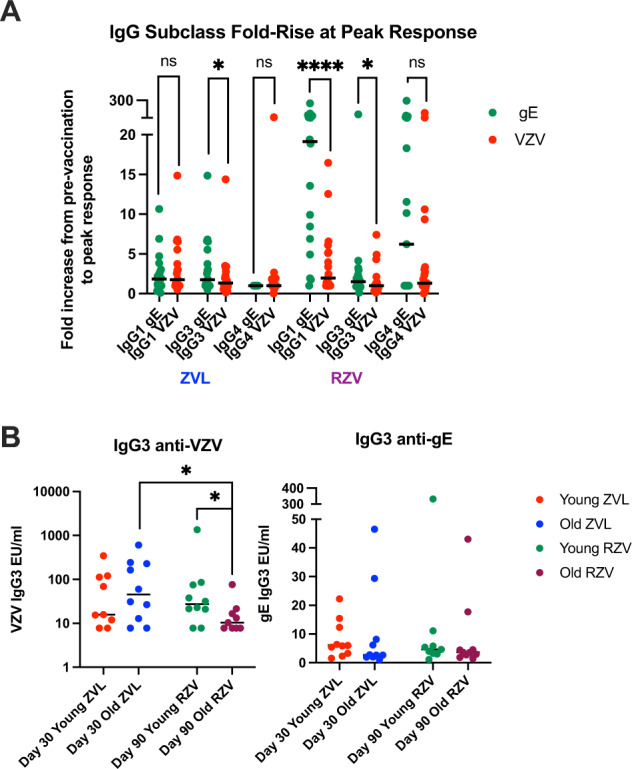
IgG subclass antibody responses to ZVL and RZV. Data were derived from 20 ZVL and 19 RZV recipients. Each symbol represents a study participant. Lines represent medians. Panel **A** shows IgG fold-increases from pre-vaccination to peak response. *Paired comparisons* of IgG1, IgG3 and IgG4 anti-gE (green) and anti-VZV (red) responses measured by Wilcoxon signed rank test (nonparametric) are shown on the graph. Panel **B** shows IgG3 anti-VZV (left) and anti-gE (right) peak responses. Significant differences between age and vaccine groups performed by Mann-Whitney test (nonparametric) are shown on the graph. * indicates 0.01 < *p* < 0.05; **** indicates *p* < 0.001.

To define the differences between anti-gE and anti-VZV ADCC responses to vaccination, we measured the IgG subtypes composing the antibody response. IgG3 antibodies are deemed to be most effective in mediating ADCC due to their long hinge that allows optimal flexibility to accommodate both target and effector cells^21^. Together with IgG1 and IgG4, IgG3 is a major component of the responses to varicella (primary VZV infection), to HZ (reactivation infection), and to varicella vaccination^22,23^. Before and after HZ vaccination, anti-VZV and anti-gE IgG1, IgG3 and IgG4 were similar in recipients of each vaccine and both age groups (not depicted). Figure 3A shows that anti-gE IgG3 fold-rise was significantly higher than anti-VZV IgG3 fold-rise after administration of ZVL or RZV assessed by paired analysis. In contrast, gE- and VZV-specific IgG1 and IgG4 responses to vaccination did not differ in ZVL recipients. In RZV recipients IgG1 anti-gE fold-rise was significantly higher than IgG1 anti-VZV fold-rise, whereas IgG4 fold-rises against gE and VZV did not differ. The analysis of IgG3 antibody levels showed that older RZV recipients had lower peak anti-VZV responses compared to younger RZV and to older ZVL recipients **(**Fig. 3B**)**. There were no significant differences in IgG3 anti-gE responses by age or vaccine group.

### gE-ADCC strongly correlates with antibody avidity

**Fig. 4:**
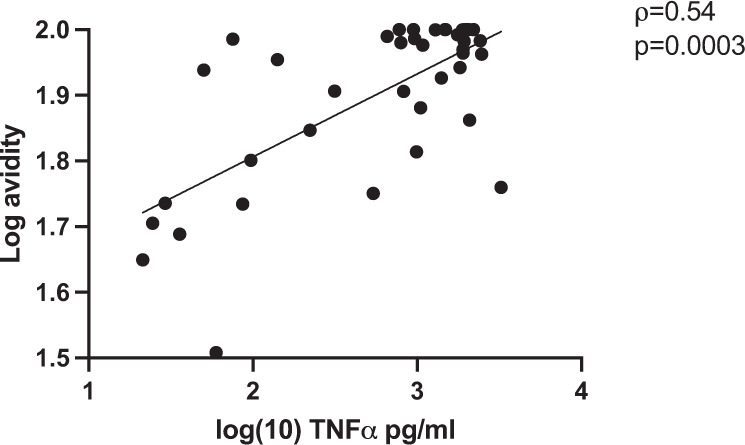
gE-ADCC correlates with anti-gE antibody avidity. Data were derived from 40 participants who received RZV or ZVL and whose ADCC responses were measured in this study. Avidity measures were previously described^24^. TNFα concentrations and antibody avidity were log-transformed for this analysis to improve the distribution of the data points. The correlation coefficient and p value were calculated using Spearman correlations.

To further characterize the antibody responses after HZ vaccination, we performed correlation analyses of VZV- and gE-ADCC measured by ELISA with anti-VZV and anti-gE antibody avidity, which we had measured in a previous study^24^. gE-ADCC strongly correlated with anti-gE antibody avidity at peak response after administration of either HZ vaccine (*ρ* = 0.5454, *p* = 0.0003; Fig. 4). Moreover, the increase from pre- to postvaccination in gE-ADCC also strongly correlated with the increase in gE avidity (*ρ* = 0.5392, *p* = 0.0003; not depicted). gE-ADCC weakly correlated with neutralizing antibody titers (*ρ* = 0.28, *p* = 0.08; Supplementary Fig. 2). In contrast, VZV-ADCC did not correlate with the avidity of anti-VZV antibodies or neutralizing titers irrespective of the type of vaccine or age.

### gE-ADCC is characterized by increased CD107α and IFNγ in addition to TNFα expression

**Fig. 5:**
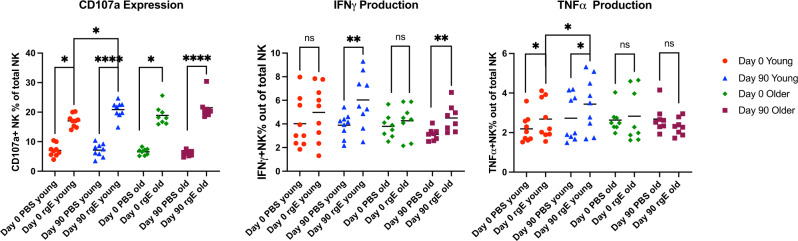
NK Functionality after Binding to gE-Specific Antibodies in RZV Recipients. Data were derived from 9 young and 8 older RZV recipients. Graphs show proportions of NK cells expressing the marker indicated in the title of each panel. Bars represent medians and quartiles. Significant differences are shown on the graph and marked by *0.01 < *p* < 0.05; **0.001 < *p* < 0.01; or ***p < 0.001.

To determine the functional characteristics of NK cells activated by the anti-gE antibodies, we investigated CD107a, IFNγ, and TNFα expression by flow cytometry in NK cells exposed to solid phase-bound gE incubated with sera from 9 younger and 10 older RZV recipients (Fig. 5). Before and after vaccination, gE-specific antibodies elicited *CD107a expression in NK cells* from both younger and older adults, but significant increases in post-vaccination responses compared to pre-vaccination responses were observed only in younger adults. RZV administration generated anti-gE antibodies in younger and older adults that stimulated *NK cell IFNγ production* that was not detected pre-vaccination. *TNFα production in NK cells* stimulated with anti-gE-specific antibodies was detected with plasma only from younger adults pre- and post-vaccination. Moreover, RZV vaccination significantly increased the ability of anti-gE antibodies to elicit NK TNFα responses in younger adults.

## Discussion

ADCC is a potential mechanism of protection against viral infections, including influenza, dengue, HIV and SARS CoV2^25–29^. We showed that administration of RZV and ZVL to adults ≥50 years of age increased ADCC elicited by antibodies against VZV lysate and gE. This was the first study of ADCC antibodies generated by HZ vaccines. Previously, studies in children with varicella detected ADCC antibodies, which preceded the appearance of neutralizing antibodies in the blood compartment, suggesting a role for ADCC in viral clearance^30^. After varicella vaccination ADCC antibodies were also observed in children who never developed neutralizing antibodies^30^.

The clinical significance of ADCC antibodies was clearly shown after RV144 vaccination for HIV, where the levels of antibodies directed toward the anti-HIV V3 loop that mediated ADCC were associated with protection^31^. ADCC antibodies are also elicited by influenza vaccines, but their protective role has not been established^32,33^. ADCC antibody responses to HZ vaccines likely play a role in protection against HZ by clearing infected cells. The comparison of RZV and ZVL showed that both vaccines increased ADCC antibodies. However, significant increases in anti-VZV ADCC antibodies post-vaccination were found only in older RZV recipients. In addition, in young adults, RZV generated higher anti-gE ADCC antibodies than did ZVL, suggesting greater ability of RZV to generate ADCC antibodies.

A tantalizing observation was the higher increase in anti-gE than anti-VZV ADCC antibodies, both after ZVL and RZV compared with pre-vaccination levels. Since IgG3, due to its long Fc hinge, is the main IgG subtype that mediates ADCC, we hypothesized that anti-gE antibodies contained higher proportions of IgG3 compared to total anti-VZV antibodies^21^. This was a novel hypothesis since the IgG subclass response to HZ vaccines had not been described. Our experiments showed that both HZ vaccines generated higher increases in anti-gE IgG3 antibodies than anti-VZV IgG3 antibodies. This difference was unique to IgG3 and did not extend to IgG1 or IgG4. We do not know if there are clinical consequences of the difference in anti-gE and anti-VZV ADCC antibodies. However, it is conceivable that this property of the anti-gE antibodies contributes to the superior protection conferred by RZV^34,35^. We previously showed that RZV generates superior cell-mediated immunity, neutralizing antibody titers, and antibody avidity compared to ZVL and here we add that greater amounts of ADCC antibodies are generated after RZV^36,37^.

Another novel observation in this study was the positive correlation between the magnitude and relative increase of gE-ADCC and anti-gE antibody avidity after vaccination. This observation expands our previous findings that anti-gE antibody avidity correlated with anti-VZV neutralizing antibodies^24^. Collectively, these data underscore the critical contribution of the physical characteristics of the antibodies to their functionality.

Interestingly, we did not observe appreciable associations of VZV-ADCC with anti-VZV antibody avidity. Moreover, the increase in VZV-ADCC or anti-VZV antibody avidity post-vaccination was more modest. There are several potential explanations for this phenomenon, which are not mutually exclusive: (1) before vaccination, VZV-ADCC, similar to avidity were already close to peak, whereas gE-ADCC and avidity were below their peak capacity to expand in response to vaccination; (2) gE contains more ADCC antibody binding sites per molecule than the average contained in VZV glycoproteins; and (3) the purified recombinant gE preparation used in our assays exposed antibody binding sites better than the inactivated whole virus VZV preparation.

We analyzed the effect of age on ADCC antibodies because older age is associated with decreased efficacy of many vaccines, including ZVL^19^. Although previous studies showed similar total IgG responses to HZ vaccines in younger and older adults, we observed a significant effect of age on ADCC, consisting of lower levels of VZV-ADCC antibodies in older compared with younger adults pre- and post-administration of either HZ vaccine. Thus, older age had similar effects on ADCC and vaccine efficacy. The investigation of IgG3 anti-VZV or anti-gE responses between younger and older adults pre-vaccination did not reveal any differences, while post-vaccination only RZV recipients showed higher levels of anti-VZV IgG3 in younger than older adults. Thus, the levels of IgG3 did not correlate with the difference in ADCC magnitude between younger and older vaccine recipients. Additional information about the physicochemical properties of the antibodies generated by the vaccines in different age groups may be required to define the age effect on ADCC. For example, the pattern of antibody glycosylation was shown to play a role in ADCC, whereby afucosylated antibodies were most likely to elicit ADCC^38^. Adjuvants may also affect the pattern of antibody glycosylation generated by vaccines^39^. In addition, our observations should be expanded with studies of other vaccines to determine if lower ADCC responses to vaccination contribute to the lower efficacy of vaccines in older than younger adults.

Our study was limited by the relatively low number of participants for the comparison of responses in younger versus older adults. Because chronological and biological age may differ in the geriatric population, hundreds of participants may be necessary to tease out the effect of age. The strength of our study was that participants were randomized to ZVL and RZV in the parent study. We also developed a new ELISA assay for ADCC based on the correlation with NK activation examined by flow cytometry. This assay is technically easier to perform and is more sensitive than the flow cytometry assay.

In conclusion, both zoster vaccines generated robust anti-gE antibodies, including IgG3. The increase in gE-ADCC post-vaccination may contribute to the protection conferred by ZVL and RZV in all age groups.

## Methods

### Study participants

For assay development, we used a convenience sample of sera with known VZV-seropositive or negative status. The effect of vaccination was evaluated using archived samples from 40 participants in a previously published study (NCT02114333)^20^. The study was approved by the Colorado Multiple Institution Review Board. All participants signed informed consent. All participants had prior varicella or had resided in the US at least 30 years, and none had prior HZ. Twenty participants were 50–59 years old; 10 received ZVL and 10 received RZV. The remaining 20 participants were 70–85 years old and were also equally divided between ZVL or RZV recipients. We used samples collected before vaccination in all participants, 30 days after ZVL administration, and 30 days after the second dose of RZV (day 90).

### Antibody-dependent cellular cytotoxicity (ADCC) measured by flow cytometry

Adapted from Gonzalez-Gonzalez et al., Immulon II 96-microwell ELISA plates were coated overnight either with VZV antigen obtained from VZV Oka strain-infected human lung fibroblasts at a multiplicity of infection of 1, harvested and UV-irradiated after 4 days of culture (VZV); similarly treated mock-infected lung fibroblasts (mock) diluted 1:20 in PBS; recombinant gE glycoprotein at 1ug/ml in PBS (rgE; GlaxoSmithKline); or PBS control. After overnight incubation at 4 °C, plates were washed and blocked with 5% fetal calf serum in PBS for 2 h at room temperature. Plasma or serum samples at the pre-optimized dilution of 1:100 in PBS were added at 100 µl/well to duplicate wells for 2 h at room temperature and plates were washed with PBS containing 0.5% Tween. PBMC from a Leukopack dedicated to this research were added at a preoptimized concentration of 200,000 cells/well in 200 µl of RPMI 1640 (Corning) containing 10% fetal bovine serum (FBS; Gemini Bio-Products), 1% glutamine (Gemini Bio-Products), 1% penicillin-streptomycin (Gemini Bio-Products), and HEPES buffer (Corning). After overnight incubation at 37 °C in a humidified 5% CO_2_ atmosphere, Brefeldin A (MilliporeSigma, 5 μg/ml), Monensin (MilliporeSigma, 5 μg/ml), and anti-CD107a (Ax488; Clone H4A3; Biolegend 328610; 1:10 dilution) were added for the last 4 hours. At the end of the incubation, PBMCs were removed, washed and stained with Zombie Yellow Viability Stain (BioLegend 423104; 1:100 dilution); antibodies against CD3 (PE-CF594; Clone UCHT1; BD 557943; 1:33 dilution), CD19 (PE-CF594; HIB19; BD 562294; 1:50 dilution), CD14 (PE-CF594; Clone MφP9; BD 562335; 1:33 dilution), granzyme B (PerCP-Cy5.5; Clone QA16A02; Biolegend 372211; 1:25 dilution), CD56 (PE-Cy7; Clone HCD56; Biolegend 318318; 1:50 dilution), CD16 (Ax700; Clone 3G8; BD 557920; 1:25 dilution); HLA-DR (APC-H7; Clone G46-6; BD 561358; 1:50 dilution), CD107a (Alexa 488; Clone H4A3; Biolegend 328610; 1:10 dilution), and perforin (BV421; Clone dG9; Biolegend 308122; 1:50 dilution). Then cells were fixed with FACS Lysing Solution (BD) and permeabilized with Permeabilizing Solution 2 (BD) and intracellular staining was performed with antibodies against granzyme B (PerCP-Cy5.5; Clone QA16A02; Biolegend 372212; 1:50 dilution), TNFα (APC, Clone Mab11; BD 551384; 1:50 dilution), perforin (BV421; Clone dG9; Biolegend 308122; 1:50 dilution) and INFγ (PE; Clone P2G10; BD 559812; 1:25 dilution). PBMC (≥200,000 events) were acquired with the Gallios instrument (Beckman Coulter). Analysis used FlowJo (Becton Dickinson) software. Gating strategy to identify activated NK cells by expression of TNFα in the ADCC assay is shown in Fig. 1A.

### ADCC assay using ELISA-measured TNFα production

The assay was performed as previously described with modifications^40,41^. PBMC stimulation was performed in Immulon II plates coated with relevant antigens as described above, at 50,000 cells/well. After overnight incubation cells were lysed with 1% v/v TritonX-100 solution in 150 mM NaCl and 20 mM Tris 7.5. Supernatants were collected and assayed for the presence of TNFα using a commercial ELISA kit as per manufacturer’s instructions (R&D Systems, Minneapolis, MN).

### IgG subclass characterization

96-well strips (Immulon 2HB; Thermo) were coated with 100μL gE at 1μg/mL (courtesy of GlaxoSmithKline) or VZV Oka strain lysate at 1:40. Plates were incubated at 4 °C overnight with 500 rpm shaking. Wells were washed 4x with PBS and blocked with 200μL PBS/.05% Tween-20/5% FBS for 2 hours. Wells were washed 4x with PBS/.05% Tween-20. Plasma samples were diluted in PBS/.05% Tween-20/5% FBS at the following preoptimized ratios: IgG1 assay 1:100; IgG3 assay 1:10; IgG4 assay 1:50. 100 μL sample/well was incubated for 2 hours at room temperature on a shaker at 500 rpm. After washing wells 4x, the following detection antibodies diluted in PBS/.05% Tween-20/5% FBS were added: Mouse anti-human IgG1 hinge-AP (Southern Biotech) 1:2000; Mouse anti-human IgG3 hinge-AP (Southern Biotech) 1:500; Mouse anti-human IgG4 Fc-AP (Southern Biotech) 1:1000. Wells were washed 4x and 100 μL pNPP 1-component AP substrate (ImmunoChemistry Technologies) were added. Color development was read at 405 nm on a MultiSkan FC (Thermo) using SkanIt software. Results were interpolated using Prism (Graph Pad).

### Statistical analysis

Statistical analyses and graphs were performed using Graphpad Prism 9 (GraphPad Software Inc., La Jolla, CA). Analyses were performed using nonparametric tests. A two-tailed *P* value of <0.05 defined statistical significance.

### Reporting summary

Further information on research design is available in the Nature Research Reporting Summary linked to this article.

## Supplementary information

Supplemental material

Reporting Summary

## Data Availability

The datasets generated and analyzed in this study will be made available to other investigators following submission and approval of a Material Transfer Agreement by the University of Colorado Tech Transfer Office.
