# Supplementary material for: Development of antibody-dependent cellular cytotoxicity in response to recombinant and live-attenuated herpes zoster vaccines
Source: NPJ Vaccines. 2022 Oct 25;7:123. doi: 10.1038/s41541-022-00545-2 (PMC9593992; doi:10.1038/s41541-022-00545-2)
Supplement: Supplementary file 1 — Supplemental material [file 41541_2022_545_MOESM1_ESM.docx]

**Supplementary Figure 1. Solid phase bound VZV antigen complexed with anti-VZV antibodies does not increase TNFα production by monocytes or B and T cell lymphocytes**. Data points indicate the proportions of TNFα-secreting T and B cells (dump) and monocytes (mono) in wells coated with VZV antigen/wells coated with mock-infected control antigen using sera from VZV seropositive (VZV+) or VZV-seronegative (VZV-) donors. Statistical analysis was performed by Mann-Whitney test.

**Supplementary Figure 2. Correlation analysis of gE-ADCC and anti-VZV neutralizing antibody titers.** Data were derived from 40 participants who received RZV or ZVL and whose ADCC responses were measured in this study. Neutralizing antibodies measures were previously published ^24^. TNFα concentrations and the reciprocal of the neutralizing antibody titers were log transformed for this analysis to improve the distribution of the data points. The correlation coefficient and p value were calculated using Spearman correlations.
